# Multiple myeloma in Latin America: A systematic review

**DOI:** 10.1002/jha2.905

**Published:** 2024-06-10

**Authors:** Vania Tietsche de Moraes Hungria, Camila Peña, David Gómez‐Almaguer, Humberto Martínez‐Cordero, Natalia Paola Schütz, Vivian Blunk

**Affiliations:** ^1^ Discipline of Hematology and Oncology Faculdade de Ciências Médicas da Santa Casa de São Paulo São Paulo Brazil; ^2^ Department of Hematology, Hospital del Salvador CICA Oriente Universidad de Chile Santiago Chile; ^3^ Department of Hematology, Hospital Universitario “Dr. José Eleuterio González” Universidad Autonoma de Nuevo Leon Monterrey Mexico; ^4^ Department of Hematology Instituto Nacional de Cancerología Hospital Militar Central de Colombia Bogotá Colombia; ^5^ Department of Hematology Hospital Italiano de Buenos Aires Buenos Aires Argentina; ^6^ Oncology Emerging Markets Pfizer São Paulo Brazil

**Keywords:** antibody therapy, epidemiology, myeloma

## Abstract

The incidence of multiple myeloma (MM) has surged globally, particularly in Latin American countries, and is attributable to an aging population and increased life expectancy. This systematic review analyzes the epidemiology, patient characteristics, and treatment outcomes for MM in selected Latin American countries: Brazil, Mexico, Colombia, Argentina, Chile, Peru, and Uruguay. PubMed and the Latin American and Caribbean Health Sciences Literature (LILACS), conference abstracts (between June 2019 and June 2022), and GLOBOCAN registry (January 2010 to June 2022) were electronically searched. Qualitative analysis employed the Joanna Briggs Institute's critical appraisal tool. Among the 586 screened articles, 26 met the inclusion criteria. The participants’ median age ranged from 54 to 67 years. GLOBOCAN data revealed that for MM, Brazil and Uruguay had the highest and lowest incidence, 5‐year prevalence, and mortality, respectively. Immunoglobulin G was the most common subtype detected. Stage III was frequently diagnosed. Though many approved drugs are available and bispecific antibodies hold promise as a future therapy, limited access, especially for CAR‐T cell‐based therapy remains a concern. The incidence of MM is increasing in Latin America. Resource constraints and costs hinder access to novel drugs and regimens. Understanding disease patterns and patient characteristics is vital to improve MM management in these countries.

## Graphical abstract

This systematic review focuses on multiple myeloma (MM) in Latin American countries, highlighting a rising trend in incidence attributable to an increasing average lifespan and the various treatment strategies currently being employed. Although most newly approved drugs for MM treatment are available in these countries, a sizeable segment of the population still has limited access to them. The use of bispecific antibodies may be achievable in the near future; however, CAR‐T cells‐based therapy still remains far from reach for most patients. To enhance outcomes, the review advocates for national guidelines, early diagnosis, universal treatment access, awareness, and collaboration to improve MM management in the region.

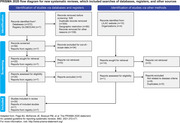

## 1 INTRODUCTION

Multiple myeloma (MM) is a hematological cancer characterized by the clonal expansion of aberrant plasma cells in the bone marrow; the condition is associated with renal damage, anemia, hypercalcemia, and osteolytic bone lesions [1, 2]. It represents 1% of all cancers and approximately 10% of all hematological malignancies [3]. According to the statistics published by GLOBOCAN in 2020, globally, there were 176,404 new cases and 117,077 deaths related to MM, of which 8.6% of the incidence and 9.6% of the mortality were from Latin America and the Caribbean [4]. The percentage change in the age‐standardized incidence rate of MM increased dramatically from 1990 to 2019 in Central Latin America (44.69%) and Tropical Latin America (46.2%) [5]. The increasing burden of MM in Latin America may be attributed to a rapidly aging population and an increased life expectancy [6]. Additionally, 90% of the patients with MM exhibit osteolytic lesions and systemic osteopenia during the disease course and nearly 60% of MM patients develop pathological fractures [7]. Likewise, patients with MM also experience renal complications with about 50% of the patients developing acute or chronic renal impairment during the disease course [8].

Autologous stem cell transplantation (ASCT) is the gold standard for treating patients with newly diagnosed MM (NDMM) [9]. Currently, MM can be treated with various drugs, including immunomodulatory drugs (IMiDs), proteasome inhibitors (PI), cytotoxic drugs, histone deacetylase inhibitors, nitric oxide, and monoclonal antibodies [10]. Use of CC chemokine receptor‐1 inhibitors, dickkopf‐1 antagonists, activin A antagonists, anti‐sclerostin antibodies, and transforming growth factor‐β‐activated kinase 1‐PIM2 inhibitors are other therapeutic options reported across the literature for managing MM‐related bone diseases [11]. Bortezomib‐based regimens continue to be the mainstay for the management of myeloma‐related renal impairment with additional therapeutic options comprising immunomodulating drug‐based regimens, PI‐based regimens, autologous stem cell transplants, and kidney transplantation for end‐stage renal disease [12].

Despite the rising incidence rates of MM in Latin America, there is a paucity of data on its prevalence, treatment, and treatment outcomes. This systematic review aims to estimate the prevalence and incidence of MM and review the treatment trends and clinical outcomes of the treatments. This study also evaluates the distribution and treatment trends of MM with regard to specific subgroups (age, gender, presence of comorbidities, disease stage, and molecular subtypes) in selected countries of Latin America, namely, Brazil, Mexico, Colombia, Argentina, Chile, Peru, and Uruguay, to fill existing knowledge gaps.

## 2 METHODS

Registration with the International Prospective Register of Systematic Reviews (PROSPERO) was performed for the protocol of this study. The systematic literature search in this review was performed in accordance with the Preferred Reporting Items for Systematic Reviews and Meta‐Analyses (PRISMA) guidelines.

## 3 OBJECTIVES

To estimate the incidence and prevalence of patients with NDMM and refractory MM in selected countries of the Latin American region (Brazil, Mexico, Colombia, Argentina, Chile, Peru, and Uruguay). To characterize the distribution of MM and the various treatments/regimens used in various subgroups of the population in selected countries of the Latin American region.

To assess the clinical and demographic characteristics of patients with MM in selected countries of the Latin American region between 2010 and 2022.

### 3.1 Search strategy

A search was performed on databases such as PubMed and the Latin American and Caribbean Health Sciences Literature (LILACS) to identify articles published between January 2010 and June 2022. The search terms used to search for relevant articles in the PubMed database are mentioned in Figure S1.

For each of the research questions, the strategy to search for relevant articles in the PubMed database was: (1) identifying the medical subject headings (MeSH) and free text words relevant to the question, (2) logically constructing search strings using MeSH and text words related to the question with the assistance of Boolean operators, (3) documenting and reviewing the search results, and (4) screening the results based on the predefined inclusion/exclusion criteria to obtain relevant articles.

The study designs considered for the literature search were limited to those cited in the eligibility criteria. The geographical settings were limited to selected countries of the Latin American region, namely, Brazil, Mexico, Colombia, Argentina, Chile, Peru, and Uruguay. Only articles written in English or Spanish were included. There were no restrictions on the ethnic status of the participants in the study. A similar search strategy was followed for the identification of relevant articles published in Spanish in the LILACS database. The latest epidemiologic data on MM from local registries and GLOBOCAN were considered. In addition, search terms similar to the ones used for the PubMed database were used to search for relevant original abstracts presented at the annual meetings of the American Society of Clinical Oncology (ASCO), American Society of Hematology (ASH), and European Society for Medical Oncology (ESMO) and published on the conference websites between June 2019 and June 2022.

### 3.2 Inclusion criteria

This systematic review includes studies involving adult patients (>18 years) diagnosed with MM. Original research studies covering randomized controlled trials, prospective and retrospective cohort studies, observational studies, case‐control studies, and cross‐sectional studies were included. Abstracts from the conference proceedings of ASCO, ASH, and ESMO identified via the above‐mentioned search strategy were also included. Articles published in English and Spanish were included.

### 3.3 Exclusion criteria

Case series, case reports, pharmacokinetic studies, and news articles were excluded. Studies on patients with MM and concomitant amyloidosis, therapy‐related MM, secondary MM, and smoldering MM were also excluded.

### 3.4 Data collection

Authors (V.B. and C.P.) independently screened the titles and abstracts of the search results. Duplicate results were removed. Thereafter, two independent reviewers thoroughly checked the full text of each article to ascertain which studies could be used for data extraction. Differences and/or conflicts were resolved mutually through discussion. The review protocol was registered in the PROSPERO database under the reference number CRD42022339932.

### 3.5 Data extraction

A Microsoft Excel spreadsheet was used to classify all the studies included in this review based on the following: (1) first author name; (2) title of the study; (3) reference details; (4) study center, place, and region; (5) study objective(s); (6) study design; (7) study period; (8) overall description of the patient population; (9) number of patients with MM; (10) number of patients with NDMM/relapsed MM/refractory MM/triple‐refractory MM; (11) age‐wise distribution of patients; (12) gender‐wise distribution of patients; (13) subtype‐wise distribution of patients; (14) comorbidity‐wise distribution of patients; (15) incidence of MM; (16) prevalence of MM; (17) treatment pattern/regimen (induction, consolidation, and maintenance regimens in NDMM cases, treatment of relapsed MM in the first relapse and second or higher relapse MM cases, and treatment regimens in transplant‐ineligible MM patients); (18) key study conclusions; and (19) article link.

### 3.6 Quality assessment

All the studies included in this review were assessed independently by two review authors (A.B. and C.D.). The Joanna Briggs Institute tool for critical appraisal of analytical studies was used to assess the quality of the eligible studies. The studies were assessed under the following domains: criteria for inclusion; study subjects and study settings; exposure measurement; measurement of condition; confounding factors; strategies to handle confounding factors; outcome measurement; and appropriate statistical analysis [13]. Each study was scored individually. The overall quality of each study was then assessed by ranking the studies. To consolidate the scores, “not applicable” was counted as a “yes,” whereas “unclear” was counted as a “no.” The sum of the points awarded to each question was divided by the highest possible score (eight) to generate a fraction (between zero and one). Scores of 0–0.3, 0.4–0.6, and 0.7–1.0 were considered low, moderate, and high quality, respectively. The review authors were not blinded to the author's information and source institution of the studies. Any disagreement was resolved by discussion or by third‐party adjudication.

## 4 RESULTS

**FIGURE 1:**
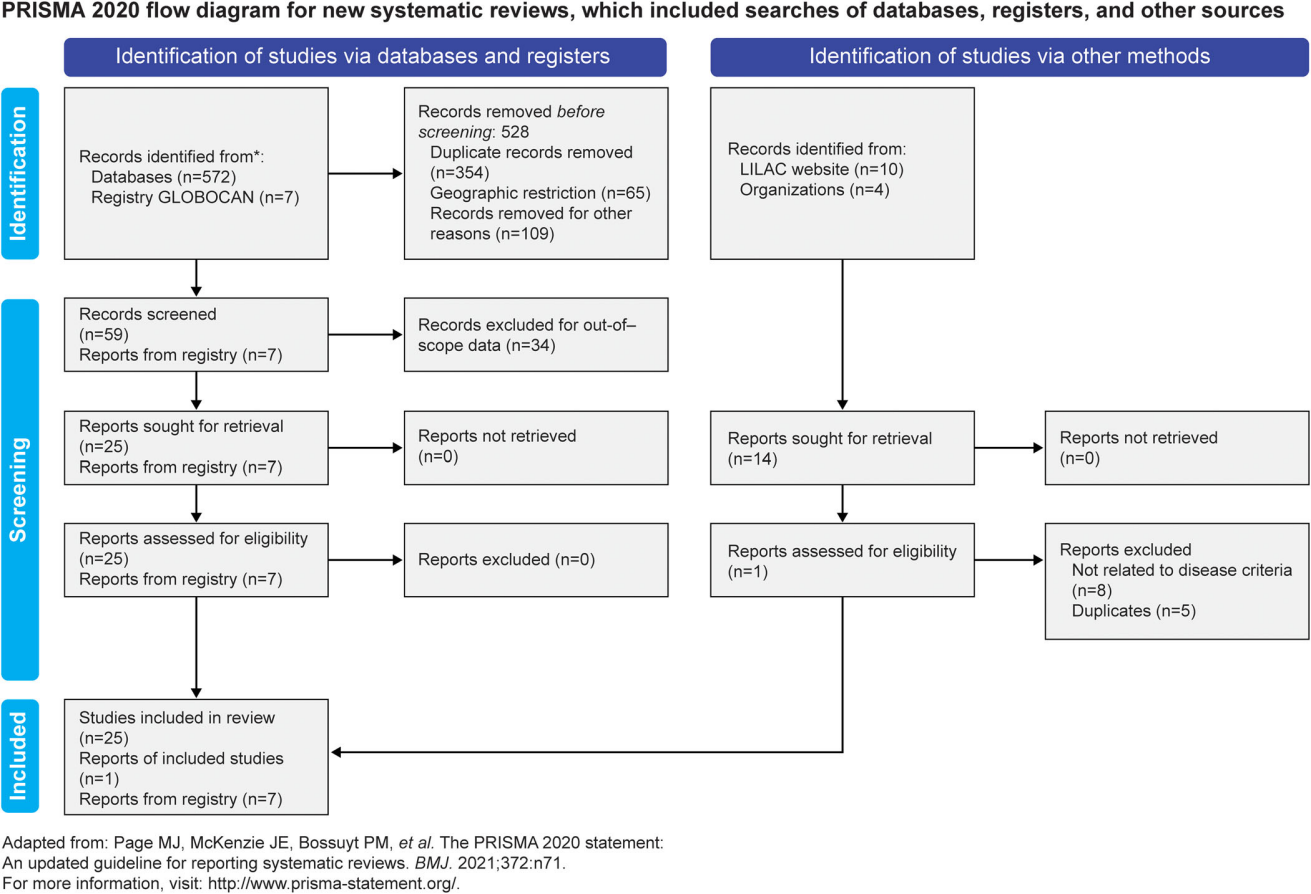
Flow diagram for study selection [14].

A total of 586 articles were retrieved from all the searches, of which 572 articles were from PubMed and 14 were from conference proceedings and the LILACS database. After the removal of duplicates, abstract/title screening, and full‐text screening, 26 studies were shortlisted based on this review's inclusion and exclusion criteria. Additionally, the GLOBOCAN registry was searched for country‐wise data, which yielded seven reports (Figure 1) [14]. The data extracted from the 26 shortlisted studies are described in Table S1 [15, 16, 17, 18, 19, 20, 21, 22, 23, 24, 25, 26, 27, 28, 29, 30, 31, 32, 33, 34, 35, 36, 37, 38, 39, 40].

### 4.1 Quality assessment

Table 1 provides the quality assessment scores of all the included studies after the application of the scoring criteria [15, 16, 17, 18, 19, 20, 21, 22, 23, 24, 25, 26, 27, 28, 29, 30, 31, 33, 34, 35, 36, 37, 38, 40, 41]. One article was not included as it was a conference proceeding. One of the 25 included articles was of “low quality” [27] and one was of “moderate quality” [26], whereas all others were of “high quality.”

**TABLE 1:** Consolidated quality assessment of included studies [15–31, 33, 34, 35, 36, 37, 38, 40, 41].

Reference	Were the criteria for inclusion in the sample clearly defined?	Were the study subjects and the setting described in detail?	Was the exposure measured in a valid and reliable way?	Were objective, standard criteria used for the measurement of the condition?	Were the confounding factors identified?	Were strategies to deal with confounding factors stated?	Were the outcomes measured in a valid and reliable way?	Was appropriate statistical analysis used?	Country/countries	Total score/8	Quality of included study
Abello et al. [15]	1	1	1	1	1	1	1	1	Colombia	1	High
Hungria et al. [16]	1	1	1	1	1	1	1	1	Argentina, Brazil, Chile, Colombia, Mexico, Panama, and Guatemala	1	High
Cowan et al. [17]	1	1	1	1	1	1	1	1	Global, North America, Asia Pacific, Latin America, Africa and East Mediterranean, and Europe	1	High
Tarin‐Arzaga et al. [18]	1	1	1	1	1	1	1	1	Peru	1	High
Linardi et al. [19]	1	1	1	1	1	1	1	1	Brazil	1	High
Jule Vasquez et al. [20]	1	1	1	1	1	1	1	1	Mexico	1	High
Etto et al. [26]	1	1	0	0	0	0	0	1	Brazil	0.4	Moderate
Balderas‐Peña et al. [21]	1	1	1	1	1	1	1	1	Brazil	1	High
Kardduss‐Urueta et al. [27]	0	0	0	0	0	0	0	1	Argentina, Colombia, and Mexico	0.1	Low
Vigolo et al. [22]	1	1	1	1	0	0	1	1	Brazil	0.75	High
Bove et al. [23]	1	1	1	1	1	1	1	1	Uruguay, Peru, Chile, Cuba, Argentina, Venezuela, and Panama	1	High
Hungria et al. [24]	1	1	1	1	1	1	1	1	Argentina, Brazil, Chile, Colombia, Mexico, Panama, and Guatemala	1	High
Peña et al. [25]	1	1	1	1	0	0	1	1	Chile	0.75	High
Peña et al. [28]	1	1	1	1	0	0	1	1	Chile, Argentina, Ecuador, Mexico, Colombia, and Uruguay	0.75	High
Crusoe et al. [29]	1	1	1	1	1	0	1	1	Brazil	0.875	High
Crusoe et al. [30]	1	1	1	1	1	1	1	1	Brazil	1	High
Hungria et al. [31]	1	1	1	1	1	0	1	1	Argentina, Brazil, Chile, Mexico, and Peru	0.875	High
Hungria et al. [41]	1	1	1	1	1	1	1	1	Latin American countries with preponderance to Brazil	1	High
Legües et al. [33]	1	1	1	1	1	1	1	1	Chile	1	High
Pena et al. [34]	1	1	1	1	1	1	1	1	Chile	1	High
Ranero et al. [35]	1	1	1	1	0	0	1	1	Uruguay	0.75	High
Vargas‐Serafin et al. [36]	1	1	1	1	1	1	1	1	Mexico	1	High
Schutz et al. [37]	1	1	1	1	1	1	1	1	Argentina	1	High
Duarte et al. [38]	1	1	1	1	1	1	1	1	Argentina	1	High
Hungria et al. [40]	1	1	1	1	1	1	1	1	Latin American countries with preponderance to Brazil	1	High

### 4.2 Study design and population

Of the 26 studies included, 16 were retrospective in nature [17, 18, 20, 22, 24, 25, 28, 29, 31, 33, 34, 35, 36, 37, 38, 40, 42], one was prospective [23], three were experimental [19, 27, 30], one was an exploratory cross‐sectional study [21], and one was a retrospective as well as a prospective study [15]. The sample sizes in these studies ranged from 29 to 26,356 patients [26, 39]. The median age of patients with MM across these studies ranged from 52.5 to 67 years. The percentages of male and female patients included in these studies varied from 45% to 100% and 0% to 55%, respectively. The median follow‐up period ranged from 2 to 101 months.

### 4.3 Epidemiology

Epidemiological data, including incidence, prevalence, and mortality were not available in the studies selected for this review. However, one study provided the standardized incidence rate of MM in Colombia in 2018 as 1.79/100,000 population and the age‐standardized mortality rate for MM in Colombia is 1.39/100,000 population [39]. The estimates for the incidence and 5‐year prevalence of MM and MM‐related mortality were gathered from the GLOBOCAN 2020 registries of the respective countries and have been summarized in Table 2. The incidence of MM ranged from 169 in Uruguay to 5655 in Brazil. The 5‐year prevalence of MM was the lowest in Uruguay at 421 and highest in Brazil at 13,568. The MM‐related mortality ranged between 132 in Uruguay and 4293 in Brazil. According to these data, Brazil has the highest incidence, 5‐year prevalence, and mortality among the Latin American countries that were selected, whereas Uruguay has the lowest [43, 44, 45, 46, 47, 48, 49].

**TABLE 2:** Country‐wise incidence and 5‐year prevalence of multiple myeloma (MM) and MM‐related mortality.

Country	Incidence	5‐year prevalence/100,000	Mortality/100,000
Argentina	1102	2835	780
Brazil	5655	13,568	4293
Chile	853	2207	652
Colombia	1376	3340	1035
Mexico	2390	5914	1538
Peru	850	2036	640
Uruguay	169	421	132

### 4.4 Clinical characteristics

Male‐to‐female ratios ranged from 0.81 [25] to 3.67 [26]. Immunoglobulin G was the most common immunoglobulin subtype detected in 28.6%–60% of all MM patients. For MM stage classification, both the International Staging System (ISS) and the Durie and Salmon system were used; however, the majority of the studies used the ISS classification. Stage III MM was the most commonly diagnosed (14.3%–66.7%) stage in MM patients. In eight studies, cytogenetic test results were available for 17.9%–32.0% of the patients [15, 28, 33, 35, 36, 37, 38, 40]. In a study by Peña et al. del17p was the most common cytogenetic variant found in 10% of all MM patients [28] In an experiment‐based cytogenetic study, abnormalities in chromosomes such as t(4;14) (p16.3;q32), del(17)(p13), and del(13)(q14) were observed in 93.5% of patients [19]. A detailed description of all the studies included in this review is shown in Table S1 [15, 16, 17, 18, 19, 20, 21, 22, 23, 24, 25, 26, 27, 28, 29, 30, 31, 32, 33, 34, 35, 36, 37, 38, 39, 40].

### 4.5 Treatment trends

A study from Colombian patients revealed that 90% of transplant‐eligible (TE) and 71% of transplant‐non‐eligible (TNE) patients received bortezomib‐based regimens as part of the first‐line therapy against MM; 47.7% of the TE patients and 22.6% of the TNE patients received cyclophosphamide–bortezomib–dexamethasone (CyBorD) therapy; 25.9% of the TE patients and 18.3% of the TNE patients received bortezomib–thalidomide–dexamethasone (VTD) therapy; and 8% of the TE patients and 17.1% of the TNE patients received bortezomib–dexamethasone (VD) therapy. Additionally, 82.4% of the TE patients and 63.2% of the TNE patients received triplet combinations, which included bortezomib or an immunomodulatory agent. Furthermore, 28.3% of the patients were consolidated with ASCT in the first‐line therapy. Most of the patients who received intensification with ASCT (70.2%) continued with maintenance therapy. Lenalidomide was the most commonly used drug (61.6%), followed by thalidomide (18.1%) and bortezomib (10.7%) [15].

A study from Argentina reported that in transplant‐eligible MM patients, although VTD induction treatment yielded higher numbers of complete responses (CRs) and very good partial responses (VGPRs), CyBorD was the preferred induction regimen in Argentina as it had a better safety profile [37].

According to the Haemato‐Oncology Latin America Observational Study (HOLA) conducted between 2008 and 2016 across the Latin American countries of Argentina, Brazil, Chile, Colombia, Mexico, Panama, and Guatemala, the first‐line therapy was largely thalidomide‐ (54.9%) and bortezomib‐based (29%). The median patient follow‐up time after the start of the first‐line therapy was 26.5 months. Compared to the first‐line therapy, the second and third‐line therapies based on thalidomide were less common [40]. It was noted that the use of bortezomib increased markedly from the period between 2008 and 2009 to the period between 2014 and 2015 (Cochran–Armitage trend test, *p* < 0.0001). The percentage of patients receiving bortezomib or bortezomib + thalidomide as first‐line treatment in non‐ASCT and ASCT patients varied from 9.1% to 13.6%, respectively, in 2008–2011 to 34.7% and 81.3%, respectively, in 2014–2015. Although the use of new agents as the first line of treatment remained limited, there was an increase in its use from 0.4% in the 2008–2009 period to 2.9% in 2014–2015. In a similar study by Hungria et al., induction chemotherapy was administered to 32.7% of MM patients before ASCT using thalidomide‐based (30.4%) and bortezomib‐based (25.2%) regimens. The thalidomide‐based chemotherapy (31.6%) and melphalan, thalidomide, and steroid therapy (16.8%) were the treatments of choice for most MM patients who had not undergone ASCT between 1998 and 2007 [24]. Furthermore, treatment regimens varied with the region. In Argentina, Mexico, Brazil, and Chile, 28%–50% of patients received thalidomide‐based chemotherapy whereas nearly 36% of these patients received bortezomib in Colombia. Transplantations were 20 times more common in Argentina than in Chile. In fact, while Latin American countries used thalidomide‐based and bortezomib‐based therapies, countries such as the United States used lenalidomide, bortezomib, and dexamethasone as frontline treatments in TE patients. Limited access and nonavailability of approved novel agents could explain the differences in treatment regimens chosen in these regions [24].

According to a retrospective study that evaluated patients diagnosed with MM between 2008 and 2012, cyclophosphamide–thalidomide–dexamethasone (CTD) regimen (28‐day cycles, with each cycle consisting of oral cyclophosphamide [400 mg/m^2^] administered for 5 days, thalidomide [100 mg] once daily in the first week and escalating to 200 mg if tolerated, and dexamethasone [40 mg once weekly]) showed good response rates (overall response rate = 69%) with an acceptable toxicity profile in NDMM patients aged ≤65 years [20]. In an exploratory cross‐sectional study conducted in Mexico between 2012 and 2014, melphalan–prednisone–thalidomide (MPT) was the most often prescribed regimen as first‐line therapy in 40% of the patients, followed by thalidomide–prednisone in 35% of the non‐transplant eligible patients. Liposomal doxorubicin–dexamethasone and MPT were the most frequently used second‐line treatments in 19% and 24% of these patients, respectively. In 32% of the patients, the third‐ and fourth‐line therapies also involved PIs [21]. Studies conducted in Brazil revealed that the VCD regimen improved the patient's condition before autologous hematopoietic stem cell transplantation (HSCT) and provided superior response rates to the treatment than the CTD regimen [22, 29]. Another study showed that between 2000 and 2016, patients with MM in Chile were treated using different therapeutic protocols such as melphalan–prednisone, vincristine–doxorubicin–dexamethasone, thalidomide–dexamethasone (taldex), and CTD. In patients who were noncandidates for HSCT, most treatments were based on MPT, although the best survival rates were observed in patients treated with CTD [25].

A study conducted between 2007 and 2011 at a public hospital in Brazil revealed that a melphalan‐ or dexamethasone‐based regimen with or without thalidomide was administered to the majority of patients (84.8%) as standard doses for induction chemotherapy. A total of 14 patients underwent ASCT after receiving high‐dose chemotherapy [19].

Another study conducted by Duarte et al. compared the efficacy of lenalidomide–dexamethasone‐based treatment in refractory or relapsed MM (RRMM) patients and reported that the combination is effective and has acceptable adverse effects [38].

Hungria et al. conducted an observational study to evaluate the pattern of care and treatment received by MM patients in public institutions in Latin American countries. The study, which included a total of 852 patients diagnosed between January 2005 and December 2007, reported that 58.6% of patients eligible for transplants received transplants, and that transplant‐ineligible patients were treated with thalidomide as first‐line therapy [31]. When the treatment trends in private (PrivC) and public (PubC) hospitals in Mexico from November 2007 to July 2016 were investigated, it was found that a thalidomide‐based regimen was the most common induction treatment used at PubCs, whereas a bortezomib‐based regimen was used most often in PrivCs [18]. A retrospective cohort study on the public and private healthcare institutions of the Grupo de Estudio Latinoamericano de Mieloma Multiple (GELAMM) countries between 2010 and 2018 found that in 1293 transplant‐eligible NDMM patients, CyBorD was the most widely used induction regimen (40%) followed by CTD (19%) and VTD (17%). However, only 53% of these patients underwent ASCT and 62% of the patients received maintenance (of these, 57% of the patients received lenalidomide‐based treatments and 33% of the patients received thalidomide‐based regimens). The study also mentions that 46% of the patients were treated in public hospitals and 54% in private healthcare facilities. Compared to the private facilities, patients treated at public institutions had more severe symptoms at diagnosis; were less frequently treated with bortezomib‐based induction therapy (41% vs. 88%; *p* < 0.001; for public vs. private hospitals), ASCT (35% vs. 69%; *p* < 0.001; for public vs. private hospitals), and maintenance therapy (54% vs. 77%; *p* < 0.001; for public vs. private hospitals); and were more frequently treated with thalidomide‐based maintenance therapy (69% vs. 14%; *p* < 0.001; for public vs. private hospitals) [28]. Detailed descriptions of these points as gathered from the studies included in this review are shown in Table S1 [15, 16, 17, 18, 19, 20, 21, 22, 23, 24, 25, 26, 27, 28, 29, 30, 31, 32, 33, 34, 35, 36, 37, 38, 39, 40].

For patients in whom PIs and IMiDs are less effective, immunotherapy has emerged as a promising treatment option. Monoclonal antibody‐based immunotherapy using naked monoclonal antibodies, antibody–drug conjugates, and bispecific T‐cell engagers and cell‐based immunotherapy using chimeric antigen receptors (CARs) to reprogram T cells are the two integral aspects of this novel approach for treating MM patients [50]. While the use of bispecific antibodies in the treatment of MM may be achievable in the near future, CAR‐T cell‐based therapy remains far from reach for the majority of the population in this region.

Crusoe et al. compared the efficacy of daratumumab in heavily treated RRMM patients in Brazil and concluded that daratumumab monotherapy in RRMM patients is both efficacious and safe. Daratumumab is an anti‐CD38 drug that induces cell death in myeloma cells [30]. The favorable safety profile and clinical activity of daratumumab monotherapy in advanced RRMM patients have also been confirmed by the phase II SIRIUS trials [51]. However, accessibility to such novel agents remains largely limited in Latin American countries.

### 4.6 Treatment outcomes

The treatment outcomes included CR, partial response (PR), VGPR, progression‐free survival, and overall survival (OS). Detailed information about the outcomes of the different treatment regimens evaluated by the studies selected for this review can be seen in Table S1.

### 4.7 Critical factors affecting treatment trends and outcomes

#### 4.7.1 Age

Data from an observational registry in Colombia show that age was independently and negatively associated with improved OS. Patients who were <65 years of age had a 5‐year OS of 70.2% (95% confidence interval [CI]: 61.2–77.5) as compared to 55.7% for older patients (>65 years of age) (95% CI: 49.3–61.6) [15]. In another study, patients who were >65 years of age at the time of diagnosis had more comorbidities and were significantly less likely to receive ASCT than patients who were <65 years (8.6% and 48.8%, respectively) [40]. According to Hungria et al., patients >65 years of age were less likely to receive bortezomib‐based or bortezomib–thalidomide as first‐line therapy (24.0%) as compared to patients <65 years of age (37.4%). However, patients >65 years of age were more likely to undergo chemotherapy or thalidomide‐based therapies [16].

#### 4.7.2 Autologous stem cell transplantation (ASCT)

Receiving ASCT treatment was also one of the factors independently associated with better OS. Patients who received consolidation with ASCT had a significantly longer median OS of 149 months as compared to 57 months for those who did not (*p* < 0.0001). Additionally, the projected 5‐year OS for patients who underwent ASCT was 80.7% as opposed to 48.7% for those who did not undergo ASCT [21]. In a study by Peña et al., patients who underwent ASCT in private hospitals had a 5‐year OS of 85%, whereas those who were treated in public hospitals had a 5‐year OS of 70% (*p* < 0.007). However, patients who did not undergo ASCT had a 5‐year OS of only 67% and 30% in private hospitals and public hospitals, respectively (*p* < 0.0001) [28].

#### 4.7.3 Type of clinic

Patients with MM treated at private hospitals had a markedly higher chance of receiving ASCT than those treated in public hospitals (49.4% and 21.6%, respectively) [40]. In addition, patients treated at public hospitals were at a higher risk of dying than those receiving care in private hospitals (hazard ratio: 2.0; 95% CI: 1.0–4.3; *p* < 0.04) [18]. When comparing outcomes in public and private healthcare settings, the 5‐year OS was 46% and 80%, respectively, with a median OS of 56 months (*p* < 0.0001) [25].

#### 4.7.4 Chromosomal abnormalities

A positive correlation was noted between the plasma cell proliferation and the proportion of cells carrying del(13)(q14); however, patients with this mutation did not have a significantly poorer survival rate than those without the mutation (*p* < 0.15). However, patients in whom >80% of the myeloma cells had the del(13)(q14) mutation had a lower predicted 3‐year OS than those in whom fewer cells had the mutation (32.4% and 69.1%, respectively; *p* < 0.033) [19]. Furthermore, the OS values of patients with t(4;14)(p16.3;q32) were significantly lower than for those without the mutation (*p* < 0.026); almost half the patients with t(4;14)(p16.3;q32) died soon after diagnosis and before initiation of treatment [19].

#### 4.7.5 MM‐related infectious complications

Diabetes mellitus (odds ratio [OR], 2.71; 95% CI: 1.23–6.00; *p* < 0.014) and an IMiD‐based regimen (particularly thalidomide) (OR, 3.02; 95% CI: 1.45–6.29; *p* < 0.003) were the variables with independent predictive significance for the occurrence of infections in MM patients [23].

## 5 DISCUSSION

The present systematic review aims to characterize the distribution of various treatment regimens for managing MM in different subgroups of people from selected countries in Latin America (Brazil, Argentina, Mexico, Chile, Peru, Uruguay, and Colombia). This study also aims to estimate the incidence and prevalence of MM and MM‐related mortality in patients with NDMM and refractory MM in these regions. An additional objective was to carry out a demographic assessment of patients with MM in these Latin American countries between 2010 and 2022. Epidemiological data are indicative of an increasing trend with regard to the incidence, disease burden, and mortality associated with MM in Latin American countries possibly due to the increasing average lifespan of the population there [6].

The findings of this review indicate that most MM patients were male (with only one study presenting the M:F ratio as 0.81 [25], around 60 years of age, and diagnosed at an advanced symptomatic stage. Stratification by age revealed that patients who were <65 years had significantly better OS than those who were >65 years of age. In addition, patients >65 years of age at diagnosis had more comorbidities and were less likely to receive ASCT than patients who were <65 years of age. This observation is consistent with the findings of a study which showed that patients who were >65 years of age had a median OS of 57.7 months as compared with a median OS of 92.72 months for patients who were <65 years of age [52].

Induction therapy with an injectable PI, an oral immunomodulatory agent, and dexamethasone, followed by treatment with autologous HSCT, and maintenance therapy with lenalidomide are the standard treatments for TE patients [2]. Most of the patients in the studies that were selected for this review received conventional treatment regimens consisting of melphalan‐ or dexamethasone‐based therapy with or without a thalidomide regimen; [19] CTD; [20, 28] MPT; [21, 42] thalidomide–prednisone; [34] CyBorD; [28, 37] CTD and VCD; [22, 29] melphalan and prednisone–thalidomide and dexamethasone–cyclophosphamide, thalidomide, and dexamethasone [25]. Crusoe et al. reported that daratumumab in heavily treated RRMM patients in Brazil was both efficacious and safe as monotherapy [30]. However, Duarte et al. and colleagues evaluated the outcomes of treatment with lenalidomide–dexamethasone on RRMM patients and found no significant differences in CR or ≥VGRP rates between treatments (*p* = 0.229), regardless of the combination received, namely, carfilzomib‐lenalidomide‐dexamethasone (KRd), Bortezomib‐lenalidomide‐dexamethasone (VRd), daratumumab‐lenalidomide‐dexamethasone (DRd), and ixazomib‐lenalidomide‐dexamethasone (IRd) [38].

However, it must be highlighted that many of the discussed treatment options in this review remain inaccessible, particularly in the public setting. Access to ASCT, particularly in the public sector, and drugs such as bortezomib is still limited despite having been the standard of care in treating MM for the last 15 years [53]. Hungria et al. reported higher OS in private hospitals in Brazil than in public hospitals and hypothesized that this finding could potentially reflect the greater availability of various therapeutic options in private settings [16].

Several prognostic factors can be used to predict the outcomes of MM. The revised version of the ISS (R‐ISS) (2015) has incorporated prognostic factors for the outcomes of MM treatment. These are chromosomal abnormalities identified by interphase fluorescent in situ hybridization (iFISH) and serum lactate dehydrogenase [54]. Cytogenetic abnormalities detected by fluorescence in situ hybridization (FISH) also have a substantial impact on therapeutic decision‐making in high‐risk MM patients [55]. However, it is important to note that only a few patients in Latin America have access to R‐ISS evaluation. Approximately 20% of the 1103 patients in the HOLA study and only 22.89% of patients in a recent study from Uruguay had the R‐ISS evaluation done at diagnosis [16, 52]. Furthermore, a recent report on access to effective diagnostic ability from the Latin American Myeloma Group (GELAMM) among 13 countries reported that iFISH was available in only 32% of public hospitals and 67% of private institutions [56]. Increased access to cytogenetic tests for patients in Latin American countries could lead to early diagnosis and timely management of MM, which could improve treatment outcomes and quality of life. However, besides the lack of access to cytogenetic testing, other factors such as lack of access to a hematologist, low awareness of the disease among general healthcare practitioners, and the vague nature of symptoms worsen the probability of an early diagnosis [57].

A high proportion of patients with MM have comorbidities that affect the clinical management of the condition. In the HOLA study, the majority of MM patients (53%) had one or two comorbidities, with diabetes mellitus and hypertension being the most common. It is important to note that 27% of all participants also had a renal disease at diagnosis [40]. Data from a Swedish Cancer Registry demonstrated that more than 50% of MM patients have comorbidities at diagnosis and that survival decreased with increasing numbers of comorbidities such as other cancers, arrhythmia, heart failure, diabetes mellitus, chronic lung disease, chronic kidney disease, and several others [58]. It should be noted that none of the included studies from Latin America (LATAM) reported on the treatment modalities specific to MM‐related bone and renal diseases.

It has also been shown that bacterial infections are a major cause of morbidity and early mortality in patients with NDMM. The use of novel anticancer agents, such as PI and IMiDs, during induction treatment has led to a greater number of infections occurring early on during therapy [23]. For this reason, antibiotic prophylaxis should be considered in patients undergoing treatment with IMiDs or PI (bortezomib), among patients with a high tumor burden, and those with a history of frequent infections or comorbidities.

## 6 STRENGTHS AND LIMITATIONS

In this systematic review, most studies were observational, leading to heterogeneity in terms of patient characteristics, treatment regimens, access to novel drugs, and follow‐up periods. Representative countries from LATAM were chosen, reasonably capturing MM distribution and treatment trends.

The studies included in this review highlight some crucial aspects related to the management of MM in this region and recommend that each country needs to formulate national guidelines for the management of MM. There is a need to focus on early diagnosis and universal access to quality treatment, increasing awareness regarding MM among people and general healthcare practitioners, generating scientific evidence, and collaborating to allow effective decision‐making and improved prognosis among MM patients [57]. Further, the paucity of data on treatment modalities specific for MM‐related bone and renal diseases highlights the unmet need in Latin America and warrants future studies directed toward the treatment of MM‐related bone and renal diseases.

## 7 CONCLUSION

There is a substantial burden of MM in Latin American countries (Brazil, Argentina, Colombia, Chile, Mexico, Peru, and Uruguay), and the incidence of this disease in these countries is on the rise. Although most newly approved drugs for MM treatment are available in these countries, a sizeable segment of the population still has limited access to them. The use of bispecific antibodies in the treatment of MM may be achievable in the near future; however, CAR‐T cell‐based therapy remains far from reach for the majority of the population in this region. The accessibility and affordability of ASCT are contributing factors to the poorer outcomes observed among patients in this region. To enhance the management of MM in these nations, specific strategies and measures to tackle the difficulties in accessibility to testing and treatment must be developed and put into action.

## AUTHOR CONTRIBUTIONS

All authors have contributed equally to the conception, design drafting, review, and finalization of manuscript.

## CONFLICT OF INTEREST STATEMENT

Vania Tietsche de Moraes Hungria: honoraria: Amgen, BMS, Celgene, GSK, Janssen, Pfizer, Regeneron, Sanofi, Takeda. David Gómez‐Almaguer: speaker/consultant: Amgen, BMS, Janssen, Novartis, Roche, Sanofi, Takeda, Teva. Vivian Blunk: employee of and own stock in Pfizer. The rest of the authors do not possess any conflicts of interest.

## ETHICS STATEMENT

The authors have confirmed ethical approval statement is not needed for this submission.

## PATIENT CONSENT STATEMENT

The authors have confirmed patient consent statement is not needed for this submission.

## CLINICAL TRIAL REGISTRATION

The authors have confirmed clinical trial registration is not needed for this submission.

## Supporting information

Supporting Information

## Data Availability

The review protocol was registered in the PROSPERO database under the reference number CRD42022339932 and is available from the corresponding author on reasonable request.
