# Supplementary material for: Multiple myeloma in Latin America: A systematic review
Source: EJHaem. 2024 Jun 10;5(4):867–78. doi: 10.1002/jha2.905 (PMC11327730; doi:10.1002/jha2.905)
Supplement: Supplementary file 1 — Supporting Information [file JHA2-5-867-s001.docx]

**LATAM Annexure I**

Supplemental Figure 1: Search strings used for the PubMed database search

| **Search strings in the PubMed database** |
| --- |
| **Incidence and Prevalence of MM**   - **Latin America**   **("Multiple Myeloma"[Mesh] OR "Multiple myeloma") AND ("epidemiology" [Subheading] OR "prevalence" OR "incidence") AND ("Latin America" OR "South America" OR "Central America") NOT ("case report") NOT ("consensus") NOT ("review")**   - **Selected countries of Latin America**   **("Multiple Myeloma"[Mesh] OR "Multiple myeloma") AND ("epidemiology" [Subheading] OR "prevalence" OR "incidence") AND ("Brazil" OR "Colombia" OR "Chile" OR "Argentina" OR "Mexico" OR "Peru" OR "Uruguay") NOT ("case report") NOT ("consensus") NOT ("review")** |
| **Treatment Trends – Newly Diagnosed MM**   - **Latin America**   **("Multiple Myeloma"[Mesh] OR "Multiple myeloma") AND ("newly diagnosed" OR "new diagnosis") AND ("Latin America" OR "South America" OR "Central America") NOT ("case report") NOT ("consensus") NOT ("review")**   - **Selected countries of Latin America**   **("Multiple Myeloma"[Mesh] OR "Multiple myeloma") AND ("newly diagnosed" OR "new diagnosis") AND ("Brazil" OR "Colombia" OR "Chile" OR "Argentina" OR "Mexico" OR "Peru" OR "Uruguay") NOT ("consensus") NOT ("review")** |
| **Treatment Trends – Relapsed/Refractory MM**   - **Latin America**   **relapsed [All Fields] OR refractory[All Fields] AND ("multiple myeloma"[MeSH Terms] OR multiple myeloma[Text Word]) AND ("Latin America" OR "South America" OR "Central America") NOT ("case report") NOT ("consensus") NOT ("review")**   - **Selected countries of Latin America**   **relapsed [All Fields] OR refractory[All Fields] AND ("multiple myeloma"[MeSH Terms] OR multiple myeloma[Text Word]) AND ("Brazil" OR "Colombia" OR "Chile" OR "Argentina" OR "Mexico" OR "Peru" OR "Uruguay") NOT ("case report") NOT ("consensus") NOT ("review")** |
| **Treatment Trends – Transplant-Eligible MM patients**   - **Latin America**   **"Transplant eligible" AND ("multiple myeloma"[MeSH Terms] OR multiple myeloma [Text Word]) AND ("Latin America" OR "South America" OR "Central America") NOT ("case report") NOT ("consensus") NOT ("review")**   - **Selected countries of Latin America**   **"Transplant eligible" AND ("multiple myeloma"[MeSH Terms] OR multiple myeloma [Text Word]) AND ("Brazil" OR "Colombia" OR "Chile" OR "Argentina" OR "Mexico" OR "Peru" OR "Uruguay") NOT ("case report") NOT ("consensus") NOT ("review")** |
| **Treatment Outcomes**   - **Latin America**   **("multiple myeloma"[MeSH Terms] OR multiple myeloma [Text Word]) AND ("treatment outcome"[MeSH Terms] OR treatment outcome [Text Word]) AND ("Latin America" OR "South America" OR "Central America") NOT ("case report") NOT ("consensus") NOT ("review")**   - **Selected countries of Latin America**   **("multiple myeloma"[MeSH Terms] OR multiple myeloma [Text Word]) AND ("treatment outcome"[MeSH Terms] OR treatment outcome [Text Word]) AND ("Brazil" OR "Colombia" OR "Chile" OR "Argentina" OR "Mexico" OR "Peru" OR "Uruguay") NOT ("case report") NOT ("consensus") NOT ("review")** |
| **Treatment Outcomes – Mortality**   - **Latin America**   **("multiple myeloma"[MeSH Terms] OR multiple myeloma [Text Word]) AND ("mortality"[Subheading] OR "mortality"[MeSH Terms] OR mortality [Text Word]) AND ("Latin America" OR "South America" OR "Central America") NOT ("case report") NOT ("consensus") NOT ("review")**   - **Selected countries of Latin America**   **("multiple myeloma"[MeSH Terms] OR multiple myeloma [Text Word]) AND ("mortality"[Subheading] OR "mortality"[MeSH Terms] OR mortality [Text Word]) AND ("Brazil" OR "Colombia" OR "Chile" OR "Argentina" OR "Mexico" OR "Peru" OR "Uruguay") NOT ("case report") NOT ("consensus") NOT ("review")** |
| **Treatment Outcomes – Survival**   - **Latin America**   **("multiple myeloma"[MeSH Terms] OR multiple myeloma [Text Word]) AND ("mortality"[Subheading] OR "survival"[MeSH Terms] OR survival [Text Word]) AND ("Latin America" OR "South America" OR "Central America") NOT ("case report") NOT ("consensus") NOT ("review")**   - **Selected countries of Latin America**   **("multiple myeloma"[MeSH Terms] OR multiple myeloma [Text Word]) AND ("mortality"[Subheading] OR "survival"[MeSH Terms] OR survival [Text Word]) AND ("Brazil" OR "Colombia" OR "Chile" OR "Argentina" OR "Mexico" OR "Peru" OR "Uruguay") NOT ("case report") NOT ("consensus") NOT ("review")** |

**Supplemental Table 1: Description of studies on patients with MM from selected countries of Latin America.^15-40^**

| **Author first name/publication year** | **Study design/study duration** | **Study population /age/gender distribution** | **Key findings** | |
| --- | --- | --- | --- | --- |
|  |  |  | **Clinical characteristics** | **Treatment regimen and clinical outcomes** |
| Abello *et al.*, 2022^15^ | Retrospective and prospective, multicenter, observational registry up to July 2020 | - N=890 real-world MM patients - Median age of 67 years (IQR, 59–75 years) at diagnosis - Age-wise groups: 44.7% of patients ≤65 years and 55.3% >65 years - 47.1% women | - Median follow-up of 18 months (IQR, 7–42 months) - ISS classification: I: 14.4%; II: 20.2%; and III: 35.1% - 58.8% of patients had DSIII classification - 36.9% of patients had IgG heavy-chain MM subtype - Cytogenetic data available for 17.9% (n=160) of patients | - 79.6% (n=709) of patients received bortezomib-based schemes as part of the first line treatment - 33.8% (n=301) of patients received CyBorD - 21.7% (n=193) of patients received VTD - 13.0% (n=116) of patients received VD - 71.8% (n=639) of patients received triplet combinations including bortezomib or an immunomodulatory agent - ORR was 79.2%,65.4%, and 59.2% for VTD, CyBorD, and VD, respectively - PR was 31.2%, 32,6%, and 23.3% and VGPR was 19%, 26.4%, and 17.2% for CyBorD, VTD, and VD, respectively - 28.3% (n=252) of patients were consolidated with ASCT in the first line of treatment - Median DFS was 62 months (95% CI: 53–75) and median OS was 88 months (95% CI: 75–106) |
| Hungria *et al.*, 2020^16^ | Multicenter, retrospective cohort study from 2008 to 2015 | - N=1103 patients with newly diagnosed MM - Median age at diagnosis was 61 years (IQR, 53–69 years) - 50.4% women | - ISS classification: I: 15.4%; II: 21.2%; and III: 31.5% - Among 221 patients with cytogenetic test results, 34 (15.4%) were found to have a high-risk cytogenetic profile [del (17p), t (4; 14), or t(14; 16)] | - 55.8% of patients were treated in public clinics and 44.2% in private clinics - First-line therapy was primarily thalidomide-based (54.9%) or bortezomib-based (29.1%) - Thalidomide-based therapy was less common as second line (24.6%) and third line (21.7%) therapy - 33.9% (n=374) patients underwent ASCT - Median PFS following first-line therapy initiation in ASCT patients was 31.1 months (95% CI: 25–36) and 15 months (95% CI: 13.4–17.1) in non-ASCT patients (log-rank p<0.0001) - Median OS following first-line therapy initiation in ASCT patients was 79.3 months (95% CI: 77–upper limit not estimable) and 52.8 months (95% CI: 46.3–68.6) in non-ASCT patients (log-rank p<0.0001) |
| Cowan *et al.*, 2020^17^ | Retrospective survey between 2006 and 2015 |  |  | - Increase of 335% in autologous HCTs from 2006 to 2015 in the Latin American region - Increase of 42% and +205% in gross incidence and utilization of auto HSCT in patients aged <70 years, respectively - Increased use of autologous HCT for MM from 3.7% to 11.4% by 2015 (an absolute increase from 282 to 1228 HCTs per year) |
| Tarin-Arzaga *et al.*, 2018^18^ | Retrospective study from October 2007 to July 2016 | - N=148 patients with newly diagnosed MM | - Median follow-up was 41 months | - 77 patients admitted to a public hospital (PubC) and 71 patients treated in private health systems (PrivC) - Thalidomide-based regimen was the most common induction treatment used at PubC - Bortezomib-based regimen was used most often in PrivC - More patients underwent autologous stem cell transplantation in PrivC - 65% of patients treated in PrivC vs. 41% treated at PubC achieved VGPR (p=0.005) - Median PFS and median OS were 23 months and 51 months, respectively, for patients treated at PubC and 41 months and 79 months, respectively, for those treated in PrivC (p<0.001) - Patients in the PubC cohort experienced a higher risk of death compared with those in the PrivC cohort (HR, 2.0; 95% CI, 1.0–4.3; p=0.04) |
| Linardi *et al.*, 2012^19^ | Cytogenetic study (experimental) | - N=92 patients recently diagnosed with MM - Median age was 63.5 years (range, 36–93 years) - 51.2% of men | - 64% of patients had IgG protein, 20.2% had IgA protein, 12.3% had light chains only (kappa = 5.6% and lambda = 6.7%), and 3.4% were non-secretory. - ISS classification: I: 13.4%; II: 36.6%; and III: 50.0% - Chromosomal abnormalities t(4;14)(p16.3;q32), del(17)(p13), and del(13)(q14) were studied in 86 patients and were detected in 8 (9.3%), 6 (7%), and 40 (46.5%) of them, respectively - Median follow-up was 37.3 months (0–54.8 months) | - Patients carrying del(13)(q14) did not have lower survival than patients without del(13)(q14) (p=0.15) - Patients with more than 80% of cells carrying del(13)(q14) showed a lower OS (estimated 3-year OS 32.4% vs. 69.1%, respectively, p=0.033) - Patients with t(4;14)(p16.3;q32) showed a statistically significant lower OS (p=0.026) - Patients received induction chemotherapy with melphalan- or dexamethasone-based ± thalidomide regimen - Fourteen patients received high-dose chemotherapy followed by ASCT |
| Vasquez *et al.*, 2021^20^ | Retrospective study between April 2008 and December 2012 | - N=59 patients with newly diagnosed MM - Median age was 56 years (range, 27–78 years); 62.7% were ≤60 years - 59% of men | - ISS stage I: 55.9%; II: 20.3%; and III: 23.7% - Predominant Ig and light chain were IgG (55.9%) and kappa (62.7%) - Median follow-up was 41 months (range: 5–138 months) | - Patients received cyclophosphamide 400 mg/m^2^ for 5 days, thalidomide 100 mg once daily, if tolerated, and dexamethasone 40 mg once weekly; in 28-day cycles) (CTD) - Total response rate was 69.5%, with a low strict complete response of 1.7%, CR 5.1%, VGPR 32.2%, and PR 30.5% - Median PFS was 35 months (95% CI, 18–41) - Median of OS was 81 months (95% CI, 33–not reached) - 23 patients were eligible for ASCT but only 10 (43.5%) patients proceeded with ASCT as a consolidation after induction - Treatment-related deaths occurred in four patients (6.7%) who were aged 62, 64, 74, and 76 years |
| Balderas-Pena *et al.*, 2019^21^ | An exploratory cross-sectional study from January 2012 to December 2014 | - N=37 patients with MM - Mean age was 61.9 years (range, 32–98 years) - 51% were men | - The most frequent clinical stage DSIII and ISS was observed in 59.46% and 54% of the patients - Mean follow-up time was 35.11 months - Other comorbid conditions found were arterial hypertension (29.72%), diabetes mellitus type 2, chronic renal failure (13.5%), and previous episode of thrombosis in 3 patients (8.1%) | - Most used first-line treatment schedule was melphalan/prednisone/thalidomide in (40%) and thalidomide/prednisone (35% ) - Global health median was 66.67 and symptoms showed a median score of 22.22 - PD was shown by 14 (38%) patients, whereas 6 (16%) patients showed CR |
| Vigolo *et al.*, 2017^22^ | Retrospective study between January 2010 and March 2015 | - N=43 patients with MM; n=29 were treated with the CTD protocol and n=14 with the VCD protocol - Mean ages of patients treated with the CTD and VCD protocols were 57.1 years and 53.6 years, respectively - 67.4% were men | - 87.8% of the patients were classified as stage III by DSIII, whereas 66.7% were stage III in the ISS - Presence of most comorbidities were similar in CTD and VTD groups, but kidney failure was more common in the VCD group (7/14 patients), compared to the CTD group (3/29 patients, p=−0.007) | - 78.3% and 48.3% of patients treated with the VCD and CTD protocols underwent ASCT, respectively - In patients given the VCD protocol, 45.5% had CR before ASCT; among those given CTD, only 7.1% had CR (p=0.023) |
| Bove *et al.*, 2022^23^ | Prospective cohort study between June 2019 and May 2020 | - N=248 patients with newly diagnosed MM - Median age was 64 (IQR, 25–90 years) | - ISS classification: I: 31.5%; II: 28.2%; and III: 40.3% - Comorbidities observed were diabetes mellitus (15.7%), respiratory disease (5.2%), and cardiac disease (4.0%) - 35.9% (n=89) patients had infectious complications with a median time to first infection of 2 months from diagnosis (range, 1–6 months) - Compared to patients without infections, patients having infections had more advanced DSIII (86.5% vs. 74.2%, p=0.023) and ISS stage III (51.7% vs. 34%, p=0.006), history of smoking (28.1% vs. 13.1%, p=0.004) and diabetes mellitus (24.7% vs. 10.7%, p=0.004) | - Highest mortality rate related to infections were seen in patients treated with IMiDs (12.5%, p=0.001) compared with PIs (2.2%) and PIs plus IMiDs (1%). - Across all treatment modalities, the most frequent infections were respiratory (PIs 34.1%, IMiDs 36.4%, and PIs plus IMiDs 42.9%) and urinary (PIs 34.1%, IMiDs 24.2%, and PIs plus IMiDs 31.4%) tract infections - Prognostic factors for the development of infections were diabetes mellitus (OR, 2.71; 95% CI, 1.23–6.00; p=0.014) and IMiD-based regimen (OR, 3.02; 95% CI, 1.45–6.29; p=0.003) |
| Hungria *et al.*, 2019^24^ | Retrospective, multicenter study between January 2006 and December 2015 | - N=1518 patients with MM - Median age at diagnosis was 61 years (range, 23–91 years) - 50.7% were men | - Most patients were ISS stage II (29.4%) or III (48.8%) - 53% of MM patients had one or two comorbidities and approximately 15% had more than two comorbidities - Hypertension was reported most frequently (in 36.5% of patients) - Other frequently occurring comorbidities were diabetes, heart disease, and renal impairment in 12.6%, 11.5%, and 35% of the patients, respectively | - Most frequent induction regimen was thalidomide-based chemotherapy - 32.7% (n=497) patients underwent ASCT; these patients were treated predominantly with thalidomide-based (30.4%) and bortezomib-based (25.2%) regimens with a median time from diagnosis to initial treatment of 15.0 days - 31.6% of the patients with MM who did not undergo transplantation received thalidomide-based chemotherapy or melphalan, thalidomide, and steroid treatment (16.8%) at induction |
| Pena *et al.*, 2018^25^ | Retrospective, multicenter study between 2000 and 2016 | - N=1103 patients with MM - Median age of 64.5 years (range, 27–95 years) - 55% were women | - ISS classification: I: 21%; II: 32%; and III: 47% | - Mean OS of patients receiving or not receiving thalidomide was 46 and 30 months, respectively (p<0.01) - Mean OS of patients treated before 2007 (treated with melphalan and prednisone) and between 2007 and 2012 (treated with thalidomide and dexamethasone) was 36 and 48 months, respectively - In the group starting in 2013 (treated with cyclophosphamide, thalidomide, and dexamethasone), the median survival had not been reached at 20 months of follow-up (p=0.01 for all comparisons) - ASCT was carried out in only 18% of the eligible patients - Median OS of the patients who receive an ACST had not been reached at 48 months compared with 36 months among those who did not receive ASCT (p<0.01) |
| Etto *et al.*, 2011^26^ | Questionnaire-based study | - N=29 patients with MM - Mean age was 55.4±13.3 years - 21.4% were women | - Stage III A: 18 (25.7%), stage III B: 8 (11.5%) - ISS classification: I: 14.3%; II: 12.9%; and III: 14.3% | - The questionnaire specific to cancer patients seems to be more informative than the generic Short   Form 36 Health Survey questionnaire and reflects the real benefits of ASCT in the quality of life of MM patients |
| Kardduss-Urueta *et al.*, 2018^27^ | Experimental study | - N=216 patients with plasma cell myeloma - Median age was 54 years (range, 29–75 years) - 56.9% were men |  | - Storing hematopoietic cells at 4 °C could expand auto transplants to centers where more complex technical skills and equipment are lacking and/or where cost may be an issue |
| Pena *et al*., 2020^28^ | multicenter retrospective cohort study (GELAMM) between 2010 and 2018 | - N=1293 transplant-eligible MM patients - Median age was 54 years   (range 22–65)   - 52.6% were men | - IgG was the most common isotype (57%), followed by light chains (LC) (19%) - ISS classification: II or III: 74% - FISH analysis was performed in only 32% of patients, with del17p being the most frequent cytogenetic variant found (10%) | - Most frequently used induction regimen was CyBorD (40%), followed by CTD (19%), and VTD (17%) - VGPR or better was attained in 37% of patients in the CyBorD group, 46% in the VTD group, and 36% in the CTD group - 53% of patients received ASCT - 62% of patients were administered maintenance therapy - 46% of patients   received treatment in public institutions   - median follow-up of 32 months (range 1–113) - 5-year OS was 64% (IC 95% CI: 60–67) - Median OS of 56 months vs. ND in public vs. private settings (p<.0001) |
| Crusoe EDQ *et al.*  2020^29^ | Retrospective multicentric study | - N=311 newly diagnosed MM patients - Median age was 58 years (range, 51–62 years) - 56.2% of men | - ISS Classification: I: 89; II: 108; III: 96 - Presence of comorbidities in both groups of patients | - VCD and CTD regimens were given - CR and VGPR in the VCD group were 17.1% and 54.7% - CR and VGPR in the CTD group were 5.1% and 42.8 %, respectively |
| Crusoe EDQ *et al.* 2021^30^ | Multicenter open-label trial | - N=49 - Median age was 65 years (range, 50–81 years) - 53.1% of men | - ECOG PS score: 0: 39.6%; 1:52.1%; 2: 8.3% | - Daratumumab was administered - PFS was 8.25 (95% confidence interval [CI], 5.55–17.54) months (Figure 2). The 12-month estimated PFS rate was 41.7% (95% CI, 26.9–55.9) |
| Hungria VTDM *et al.*  2017^31^ | Multicenter retrospective-prospective observational study | - N=852 - Mean age in transplant-ineligible group=67.4 - Mean age in transplant-eligible group=54.7 | - Transplant ineligible patients ISS classification: 37.9% (III) - Transplant-eligible patients ISS classification: 30% (III) | - Median OS in transplant-ineligible patient=43 months - Median OS in transplant-eligible patients=60.5 months |
| Hungria VTDM *et al*. 2019^32^ | Retrospective observational study | - N=1968 - Median age was 60.8 years (ranging from 52.6–69.3 years) - 51.5% of men | - Patients from Latin America were significantly younger and had more hypercalcemia/bone lesions than Asian patients, in whom anemia and more advanced ISS stage (stage II: 42.6%) were more common | - Median OS in patients undergoing transplant=92 months - Median OS in patients not undergoing transplant=47 months |
| Legües ME *et al.*  2019^33^ | Retrospective study | - N=30 - Median age was 54 years (range 32–76 years) - 56% men | - ISS staging III: 60% | - Genetic abnormalities seen in 47% of the patients; the t(4;14) abnormality was observed in 19% of patients, del(17p) was observed in 10% of patients, and t(14;16) was not detected |
| Pena C *et al.* 2019^34^ | Retrospective, multicenter, observational study | - N=177 - Median age was 52.5 years | - - ISS classification Stage III: 32.2 % | - OS at 5 years=70% - 100% in transplant patients - 62% in those without transplant - OS was 92 months |
| Ranero S *et al.*  2018^35^ | Observational, retrospective, descriptive study | - N=36 - Median age was 57 years (range, 37–77 years) - 50% men | - ISS classification II=77.8%; 36 patients received bortezomib. The most frequently used plan was cyclophosphamide-bortezomib-dexamethasone, subcutaneously administered in 54% of cases. | - Global response rate was 79.5% (87% in the first line, 68.8% in the second or third line). With a median follow-up of 26 months; global survival was 61% and progression-free survival was 35 months, (CI 95%, 22.6–47.4) |
| Vargas-Serafin C *et al.*  2021^36^ | Retrospective single center study | - N=245 - Median age=62 years (35–92 years) - 49% of men | ISS classification: II (58.4%)  Low SES: 83.7%  Comorbidities: 69.4% | - CR=20.1% - VGPR=44.9% - Median OS=44 months |
| Schutz N *et al.*  2020^37^ | Retrospective multicenter study | - N=322 - Median age=57 years (26–74 years) - 52% of men | ISS classification III=28%  IgG=46%  18% renal failure | - Post ASCT   VTD grp; CR=48.53   - CyBorD grp; CR=40.00 |
| Duarte PJ *et al.*  2021^38^ | Retrospective multicenter | - N=156 cases of refractory/relapsed MM - Median age=61 years (range, 34–83 years) | - Patients treated with Rd triplets, 86 (55%) patients received KRd, 30 (19%) VRd, 30 (19%) DRd, and 10 (6%) Ird;   no significant differences in terms of performance status, ISS score, subtype of paraprotein, CRAB symptoms, stem cell transplant (SCT) were found between these groups | - The PFS at 18 months was 80% (95% CI 54–92) for DRd vs. 75% (95% CI 29–93), for IRd vs. 53% (95% CI 29–73) for VRd |
| Martinez-Cordero H *et al*.  2020^39^ | Cross-sectional study | - N=26356 MM cases | - Incidence=1.79 x 100,000 - Adjusted prevalence=8.97 x 100,000 inhabitants/year - Age-standardized mortality= 1,39x100,000 |  |
| Hungria V *et al* 2020^40^ | Propensity score matching | - N=1103 newly diagnosed MM | - In HOLA grp - ASCTs were for thalidomide plus corticosteroids (32.1%) and thalidomide plus an alkylating agent (18.4%); after matching, proportions of these regimens were 23.6% and 27.9%, respectively | - In HOLA grp – after matching:   PFS=18.1 months (95% CI 15.0–23.1) vs.  In D-VMP grp (daratumumab- bortezomib, melphalan, and prednisone)   - PFS=37.7 months (95% CI 32.0–not reached) |

*ASCT: Autologous stem cell transplantation; CR: Complete response; CRAB: Calcium elevation, renal insufficiency, anemia, bone abnormalities; CyBorD: Cyclophosphamide, bortezomib, dexamethasone; CI: Confidence interval; CTD: cyclophosphamide, thalidomide, and dexamethasone; DSIII: Durie–Salmon III classification; DFS: Disease-free survival; FISH: Fluorescence in situ hybridization; HCTs: Hematopoietic cell transplantation; HR: Hazard ratio; IQR: Interquartile range; ISS: International Staging System; IgG: Immunoglobulin G; IgA: Immunoglobulin A; IMiDs: Immunomodulatory drugs; MM: Multiple myeloma; ND: Not reached; OR: Odds ratio; OS: Overall survival; ORR: Overall response rate; PR: Partial response; PD: Progressive disease; PFS: Progression-free survival; PIs: Protease inhibitors; VD: Bortezomib and dexamethasone; VCD: Bortezomib, cyclophosphamide, and dexamethasone; VGPR: Very good partial response; VTD: Bortezomib, thalidomide, dexamethasone.*
